# In Memoriam—Dr. Mary E. Grady

**Published:** 1896-07

**Authors:** 


### IN MEMORIAM—DR. MARY E. GRADY, OF BROOKLYN, a graduate of the New York Medical College and Hospital for Women, and the New York Ophthalmic Hospital College, died in Brooklyn, June 29th. She was a very talented woman and thorough oculist and specially successful in refraction work.

For two and a half years she was associated with Dr. Bushrod W. James, of Philadelphia. She was practicing ophthalmology in Brooklyn at the time of her death, which was of pulmonary disease, the result of pneumonia.

